# Nanostructured Biomaterials for Bone Regeneration

**DOI:** 10.3389/fbioe.2020.00922

**Published:** 2020-08-21

**Authors:** Joseph G. Lyons, Mark A. Plantz, Wellington K. Hsu, Erin L. Hsu, Silvia Minardi

**Affiliations:** ^1^Department of Orthopaedic Surgery, Northwestern University Feinberg School of Medicine, Chicago, IL, United States; ^2^Simpson Querrey Institute, Northwestern University, Chicago, IL, United States

**Keywords:** bone regeneration, biomaterials, nanomaterials, delivery systems, stem cells

## Abstract

This review article addresses the various aspects of nano-biomaterials used in or being pursued for the purpose of promoting bone regeneration. In the last decade, significant growth in the fields of polymer sciences, nanotechnology, and biotechnology has resulted in the development of new nano-biomaterials. These are extensively explored as drug delivery carriers and as implantable devices. At the interface of nanomaterials and biological systems, the organic and synthetic worlds have merged over the past two decades, forming a new scientific field incorporating nano-material design for biological applications. For this field to evolve, there is a need to understand the dynamic forces and molecular components that shape these interactions and influence function, while also considering safety. While there is still much to learn about the bio-physicochemical interactions at the interface, we are at a point where pockets of accumulated knowledge can provide a conceptual framework to guide further exploration and inform future product development. This review is intended as a resource for academics, scientists, and physicians working in the field of orthopedics and bone repair.

## Introduction

Bone undergoes self-repair of small defects due to the synergistic actions of mesenchymal cells, osteogenic cells, and cells of the immune system (Marsell and Einhorn, 2011). This self-repaired bone contains physico-chemical and mechanical properties that recapitulate the bone which was replaced (Dimitriou et al., 2011). However, larger defects are unable to undergo the same level of self-healing, and regenerative medicine approaches are paramount in addressing these clinical challenges (Ho-Shui-Ling et al., 2018).

Autologous and allograft bone are generally considered the clinical standard-of-care for bone repair (Grabowski and Cornett, 2013; Gupta et al., 2015), despite critical limitations such as supply and quality of host bone, donor site morbidity (Angevine et al., 2005; Gruskay et al., 2014), and immunogenicity, respectively (Stevenson and Horowitz, 1992; Bauer and Muschler, 2000). Osteoinductive growth factors, in particular recombinant human bone morphogenetic protein-2 (rhBMP-2), have demonstrated remarkable efficacy, but a number of concerns and controversies exist regarding the safety of their clinical use and high cost (Burkus et al., 2002, 2003; Carragee et al., 2011; Singh K. et al., 2014; Vavken et al., 2016; Zadegan et al., 2017). Although numerous synthetic bone graft substitutes are available, the problem of delayed and/or compromised healing remains a significant clinical challenge (Zura et al., 2016; Fernandez et al., 2020).

The ideal biomaterials for bone regeneration should not only be biocompatible and osteoconductive but also osteoinductive. They should be able to leverage the self-healing capabilities of the bone by (i) providing the main structural, compositional, and biochemical cues for the formation of new tissue; (ii) engaging the host’s resident immune cells in the regenerative response; (iii) promoting the recruitment, proliferation, and differentiation of progenitor cells; and (iv) recovering an adequate local blood supply to support healing and remodeling (Schmidt-Bleek et al., 2014; Minardi et al., 2015a).

Recently, nanotechnology has become a domain with breakthrough potential to further propel the field of bone regeneration. Nanostructured biomaterials have proven superior at enhancing bone regeneration due to their unique chemical and physical properties (e.g., magnetic, electrical) that are uniquely different from their bulk counterparts (Perez et al., 2013; Wang Q. et al., 2016). These differences stem from an ability to be engineered to precisely mimic the composition and nanoarchitecture of bone, while allowing for the recapitulation of crucial characteristics of its biochemical milieu at the nanoscale (Minardi et al., 2016b). This translates in improved ability to engage the host’s immune and progenitor cells at the nanoscale, resulting in enhanced outcomes (Cheng et al., 2013).

**FIGURE 1:**
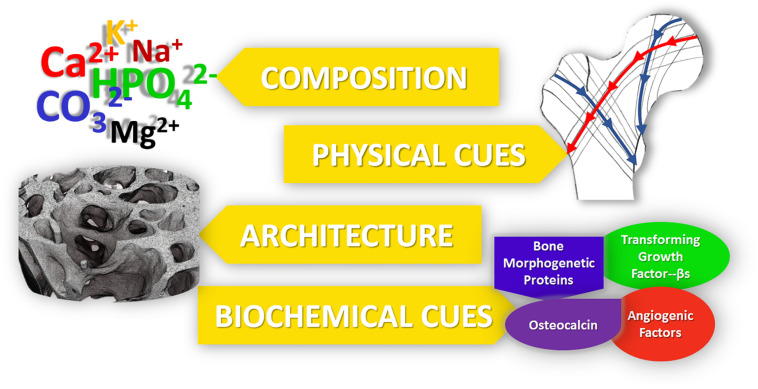
The five main properties of bone, which should be recapitulated into bone regenerative strategies for improved outcomes.

In the rational design of regenerative nanotechnologies for bone regeneration, four crucial elements of bone should be considered and recapitulated as closely as possible: (i) composition, (ii) physical stimuli, (iii) architecture and (iv) biochemical cues, as summarized in Figure 1. Inspired by mimicking these 4 fundamental characteristics of bone, a plethora of nanostructured materials have been developed over the last decade to elicit bone regeneration. Technologies that recapitulate more than one of these four fundamental elements have been shown to lead to superior outcomes. This review highlights such ongoing work in the field of nanostructured materials for bone regeneration and their potential in clinical practice.

## Mimicking Bone Composition: Bioceramics and Composite Nanostructured Biomaterials

### Bioceramics

Bone is a natural nanostructured composite, consisting of approximately 60% (dry weight) mineral, mostly nano-apatite—which is a calcium phosphate (CaP) ceramic (Minardi et al., 2015a). Accordingly, a number of bioceramics containing calcium and phosphorous have been proposed for bone regeneration (Hench et al., 2014; Jones et al., 2016). Of these, CaP materials most closely mimic the mineral phase of bone and have demonstrated relatively greater osteoinductivity, making CaP a common material of choice for bone grafts. A number of bioceramics have been used clinically for several decades (Szpalski and Gunzburg, 2002; Giannoudis et al., 2005; Campana et al., 2014; Fernandez de Grado et al., 2018), both for load- and non-load- bearing applications (Roberts and Rosenbaum, 2012). While conventional bioceramics had poor mechanical properties and unfavorable biodegradability and porosity (Fielding and Bose, 2013; Wen et al., 2017), the latest generation of bioceramics are structured at the nanoscale and have significantly improved bioactivity, biodegradation and mechanical properties, and are reviewed below. Their advantages and disadvantages are summarized in Table 1.

**TABLE 1:** Summary of the main nanostructured calcium-phosphate based materials for bone regeneration, with their respective advantages and disadvantages.

	**Advantages**	**Disadvantages**	**References**
**Nanostructured Bioceramics**			
Nano-bioglasses	Biocompatible	Suboptimal Biodegradation	Vallet-Regí et al., 2003; Izquierdo-Barba et al., 2013; Ducheyne, 2015; Islam et al., 2017; Mancuso et al., 2017
	Enhanced bone integration	Poor mechanical properties	
	Improved biodegradation		
Hydroxyapatite	High biocompatibility	Poor mechanical properties	Liou et al., 2004; Capuccini et al., 2008; Boanini et al., 2010; Tampieri et al., 2012; Zofková et al., 2013
	Resembles mineral phase of bone		
	Can be doped with multiple ions to closely mimic bone mineral	Slow degradation rates *in vivo*	
	Osteoconductive		
	Can be used in a plethora of formulations (e.g., powder, solid scaffold, cement, coatings)	Limited osteoinductivity	
	High biocompatibility		
Tricalcium phosphate	Provides main bulding blocks for new matrix deposition	Poor mechanical properties	Shepherd and Best, 2011; Sadat-Shojai et al., 2013; Vahabzadeh and Bose, 2017; Sergi et al., 2018
	Can be doped with multiple ions to tune bioactivity and degradation		
	Osteoconductive		
	Can be used in a plethora of formulations (e.g., powder, solid scaffold, cement, coatings)		
	Faster *in vivo* degradation		
**Nanocomposites**			
Ceramic/polymer composites (e.g., HA/PLGA, HA/Alginate)	High biocompatibility	May have limited osteoinductivity	Kim et al., 2005; Miao et al., 2005; Tampieri et al., 2005; Heo et al., 2009; Akman et al., 2010; Bernstein et al., 2010; Cruz, 2010; Bhumiratana et al., 2011; Wang Z. et al., 2016; Zhu et al., 2017; Bian et al., 2019
	Ease of fabrication	Fabrication requires organic solvents	
	Can be used to prepare scaffolds with complex 3D architecture		
	Improved mechanical properties of scaffolds		
	Tunable degradation rate		
Bio-hybrid composites	High biocompatibility	Poor mechanical properties (not load bearing)	Tampieri et al., 2008; Tampieri et al., 2011; Minardi et al., 2015a; Minardi et al., 2019
	Fabrication not requiring organic solvents		
	Highly biomimetic		
	Excellent bioactivity		

#### Hydroxyapatite-Based Ceramics

Among CaP ceramic phases, synthetic hydroxyapatite (HA) has been the one most extensively studied due to its biocompatibility and resemblance to the composition of natural bone mineral (Sadat-Shojai et al., 2013; Šupová, 2015). First generation materials were fabricated with stoichiometric HA [Ca_10_(PO_4_)_6_(OH)], which has been successfully synthesized and mass produced through several synthesis strategies, including hydrothermal reactions, sol–gel syntheses, and mechanochemical syntheses (Kalita and Bhatt, 2007). However, natural bone mineral is produced in a very dynamic environment with numerous ions present (e.g., Mg^2+^, K^+^, Na^+^, CO${}_{3}^{2-}$, HPO${}_{4}^{2-}$), which frequently substitute ions in the apatite lattice. The apatite present in natural bone is calcium deficient and is characterized by a Ca/P ratio lower than the typical 1.67 of stoichiometric HA (Kalita and Verma, 2010; Dziadek et al., 2017). Ion substitution plays an important role in maintaining the low crystallinity of bone apatite, which is crucial for bone metabolism. This low crystallinity may correspond to higher reactivity *in vivo*, resulting in faster bone formation and remodeling (Minardi et al., 2015a). In contrast, stoichiometric HA is more crystalline and stable in aqueous solutions, resulting in a less biodegradable material that could impede the formation of new bone through the entirety of a defect space or osteointegration with the surrounding matrix (Liou et al., 2004; Tampieri et al., 2012). To overcome these limitations, numerous biomimetic multi-substituted nano HAs have been developed to mimic the natural mineral phase of bone and enhance bioactivity and solubility (Boanini et al., 2010; Zofková et al., 2013).

Various substituted nanostructured HAs have been proposed, some of which have been used as tools to fine-tune or stimulate specific biological functions. For example, Mg^2+^ plays a vital role in osteogenesis and is present in young and newly formed bone (De Bruijn et al., 1992). Landi et al. (2006) found that Mg-substituted HA showed enhanced cell adhesion, proliferation, and metabolic activity compared to HA. Due to the smaller ionic radius of Mg^2+^ relative to Ca^2+^, the Mg-substituted structure is more unstable when incorporated into the crystal lattice (Fadeev et al., 2003). Mg is also thought to induce nitric oxide production in endothelial cells, a critical component of angiogenesis (Maier et al., 2004). Sr acts to enhance bone formation *in vivo* by inhibiting osteoclast-mediated bone resorption while upregulating osteoblast activity (Li et al., 2009; Ozturan et al., 2011), which is why Sr-based drugs have been long used to treat osteoporosis (e.g., strontium renelate; Capuccini et al., 2008). Thus, Sr-doped nano-HA has also been extensively used in bone regenerative strategies (Wong et al., 2004; Frasnelli et al., 2017; Neves et al., 2017; Ratnayake et al., 2017). Similarly, substitution with Zn has been shown to enhance osteogenic activity (Ren et al., 2009), with a proposed mechanism of inhibiting osteoclast resorption and upregulating osteoblastic activity (Hadley et al., 2010; Yamaguchi and Weitzmann, 2011). More recently, Tampieri et al. (2010) proposed a conceptually new type of nanostructured calcium-deficient HA, by substituting it with Fe^2+^ and Fe^3+^ to endow the HA with superparamagnetic properties. This magnetic behavior may potentially be exploited for bone regeneration purposes to enhance osteogenesis (Tampieri et al., 2012).

Alternatively, a common anionic substitution involves CO${}_{3}^{2-}$ replacement of the phosphate group within nano-HA, which may influence bone turnover and metabolism (Du et al., 2019). When incorporated in HA, it showed enhanced osteoconductive potential compared to pure HA (Du et al., 2019), while increasing its solubility due to its decreased crystallinity (Wang and Nancollas, 2008). In contrast to these effects, F^–^ doped HA results in decreased solubility (Kim et al., 2004) and increased strength, therefore reducing the brittleness of the CaP (Bianco et al., 2010). Si-HA showed instead improved osteoblast attachment and differentiation, and decreased osteoclast differentiation *in vivo* (Matesanz et al., 2014).

#### Tricalcium Phospohate-Based Ceramics

Another popular type of CaP ceramic used extensively in orthopedics is tricalcium phosphate (TCP). Two types of TCP have been pursued for bone regeneration: α-TCP and β-TCP. They differ in their atomic arrangements (Wen et al., 2017), but both have a Ca/P ratio of 1.5 (Wen et al., 2017). β-TCP has become the TCP of choice, given its superior rate of degradation and bioactivity over α-TCP (Kamitakahara et al., 2008; Ghanaati et al., 2010). Hydroxyapatite and TCP can also be combined in varying ratios within composite scaffolds to tune degradation and potentially enhance osteoconductive and osteoinductive properties (Daculsi, 1998; Arinzeh et al., 2005; Samavedi et al., 2013). Similar to HA, TCPs can also undergo ion-substitution as a tool to create ceramic-based materials that target specific biological pathways *in vivo*. For example, Mg-doped β-TCP and Sr-doped β-TCP-based materials have shown improved bone healing through accelerated osteogenesis and angiogenesis in a large animal model (Bose et al., 2011; Tarafder et al., 2015), with improved mechanical strength compared to the pure TCP scaffolds (Tarafder et al., 2015). Similar to Fe-doped HA, Fe-doped TCP stabilized the β-TCP phase, and osteoblasts showed enhanced cell adhesion to doped-TCP relative to pure TCP (Vahabzadeh and Bose, 2017). Moreover, cell proliferation was reportedly enhanced in TCP doped with other ions, such as Mg^2+^, Zn^2+^, Sr^2+^, and Li^+^ (Vahabzadeh and Bose, 2017).

Using these ceramic phases, numerous types of nanostructured 3D scaffolds (and bone cements) have been prepared through a variety of ways, including dry methods, wet methods, and high temperature methods (Sadat-Shojai et al., 2013). Dry methods include solid-state and mechanochemical reactions. The solid-state and mechanochemical technique have the advantage of a simple procedure for large scale production, whereas the mechanochemical technique reliably produces a specific nanostructure (Sadat-Shojai et al., 2013). Wet methods are commonly used and include techniques including but not limited to sol–gel synthesis and hydrothermal synthesis. These methods have the advantage of producing nanoparticles with a consistent morphology and size (Shepherd and Best, 2011; Sadat-Shojai et al., 2013). The downfall of these techniques is that the products can often have multiple phases present (Sadat-Shojai et al., 2013). High temperature processes such as combustion and pyrolysis are capable of bypassing the problem of multiple phases, however control over the byproducts limits this method’s applications (Sadat-Shojai et al., 2013). Moreover, there a numerous techniques to introduce porosity within 3D CaP scaffolds, including a polymeric sponge technique (Monmaturapoj and Yatongchai, 2011), foaming technique (Sopyan et al., 2007), supercritical foaming technique (Diaz-Gomez et al., 2017), gel casting of foams (Sopyan et al., 2007), and slip casting (Sopyan et al., 2007). Although all of these nanostructured ceramics are limited by poor mechanical properties, their strong osteoconductive potential makes them attractive for use as coating materials for load bearing implants, where such use may enhance osteointegration or even have antibacterial properties (Sergi et al., 2018).

Although nanostructured calcium-deficient CaP materials have provided enhanced biomimicry of the mineral phase of native bone, they have not proven capable of recapitulating all of its subtle and complex physiochemical properties. Thus, strategies based on nanostructured composites have been developed to fulfill this goal.

### Nanostructured Composites

Biomimicry is an increasingly popular strategy in regenerative medicine, aiming to engineer materials that closely resemble the target tissue (Nandakumar et al., 2013). Since bone is a natural composite—made of an inorganic component (mostly multi-substituted HA) and an organic component (mostly type I collagen)—researchers have long focused on developing nanostructured ceramic/polymer composite materials with the purpose of recreating the composition and function of natural bone. Nanostructured composites for bone regeneration leverage the osteoconductivity of synthetic CaP ceramic phases and the unique mechanical properties of polymers. For example, both synthetic polymers like poly(L-lactic acid) (PLLA; Cruz, 2010; Zhu et al., 2017), poly(e-caprolactone) (PCL; Heo et al., 2009; Bernstein et al., 2010), poly(lactic-co-glycolic acid) (PLGA; Miao et al., 2005; Wang Z. et al., 2016) as well as naturally occurring polymers such as gelatin (Kim et al., 2005), silk (Bhumiratana et al., 2011), chitosan (Akman et al., 2010), alginate (Tampieri et al., 2005), and collagen (Bian et al., 2019) have been combined with HA and TCP to fabricate a plethora of composite materials over the past three decades. These composites have been fabricated in a myriad of ways: electrospinning, gas foaming, solvent casting and particulate leaching, phase separation, and melt mixing have been widely used to fabricate scaffolds (Alizadeh-Osgouei et al., 2019). The major drawback, common to all these approaches in the manufacturing of porous structures is the inability of conventional methods to completely control the architecture of scaffolds, such as pore size and interconnections. Furthermore, the use of solvents required by some of these methods can impact scaffold biocompatibility (Alizadeh-Osgouei et al., 2019). Additive manufacturing is a new and modern technique that shows great potential to offer complete control of architectural details such as pore size, which significantly affects the properties of ceramic-based scaffolds. 3D-printing techniques have received much attention due to the capacity to fabricate specific and complex structures (further discussed in paragraph 4) (Kumar et al., 2019).

**FIGURE 2:**
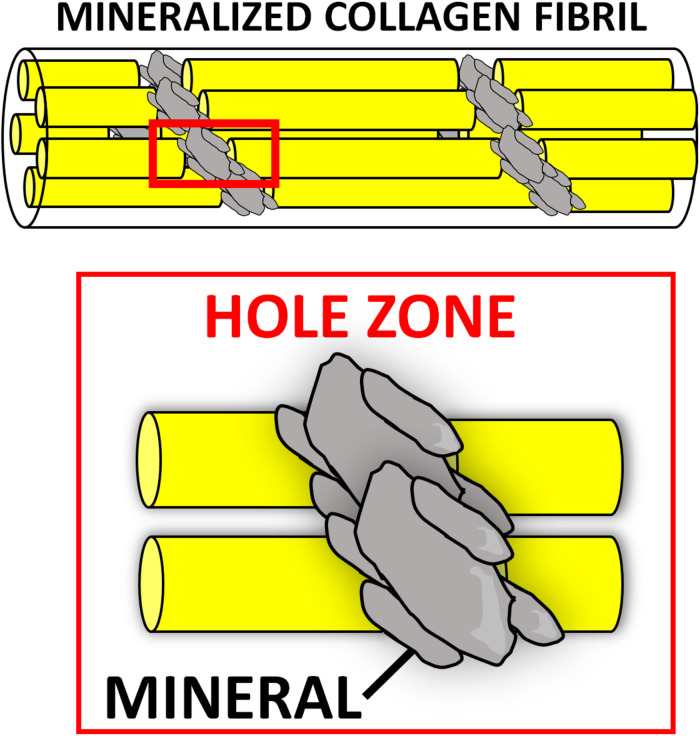
Intrafibrillar mineralization occurring during bone biomineralization. The nucleation of the mineral (gray) is thought to begin at the “hole zones” of the collagen fibrils (in red box), between single collagen molecules (yellow), as depicted in this schematic.

Numerous composite materials have been fabricated with natural polymers, with the underlying hypothesis that mimicking natural bone matrix would harness regeneration. A plethora of CaP/natural polymer composites have been described. The first generation of such composites was prepared by blending the desired ceramic phase with the natural polymer of choice in aqueous solutions (Ridi et al., 2017). Although these materials contained the two main components of bone matrix, they lacked vital chemical, physical, and topographical information at the nanoscale, which cells need to repair bone (Tampieri et al., 2011). The organic matrix (mostly type I collagen) of natural bone acts as a template for the nucleation of the mineral phase, directing its deposition, and guiding the growth of the mineral crystals along its fibers via interaction of its functional groups (e.g., carbonyl groups) with the apatite crystals. It is believed that the mineralization begins in correspondence of the hole zones of the collagen fibrils (*intrafibrillar mineralization*) (Figure 2). This highly regulated chemical-physical interaction between the inorganic and organic phase not only directs the orientation of the forming apatite crystals, but also limits their crystallinity, which is paramount to the formation of a nanocomposite material (i.e., bone extracellular matrix [ECM]) (Kim D. et al., 2018). The unique characteristics of both stiffness and flexibility of the bone result from this intimate interaction between these two components (Nair et al., 2014). Thus, several groups have focused on the development of biologically inspired synthesis methods to mineralize natural polymers by mimicking the process of bone biomineralization. In these syntheses, the ceramic phase is deposited onto the organic template during its self-assembly through a pH-driven process which resembles that of bone biomineralization. Using this synthetic approach, the mineral phase is not simply mixed with the organic template, but nucleated directly onto it and intimately bonded to the organic matrix, resulting in nanostrustured “bio-hybrid composites” (Minardi et al., 2015a). Accordingly, many studies have described the bio-inspired mineralization with nanoapatite phases of several natural polymers, such as chitosan (Palazzo et al., 2015), alginate (Tampieri et al., 2005), gelatin (Landi et al., 2008), and type I collagen (Tampieri et al., 2008). The main advantages of these materials are: (i) their ability to mimic bone matrix at the nanoscale, storing the crucial nano-compositional and topographical information necessary for cell migration, proliferation and osteogenic differentiation (Minardi et al., 2015a); (ii) their high degree of interconnected porosity, conventionally achieved by freeze-drying (Wu et al., 2010a), which facilitates cell infiltration and neovascularization (O’Brien et al., 2004); (iii) their syntheses do not require harsh conditions, allowing for the incorporation of a variety of delivery systems and bioactive molecules (Minardi et al., 2016b). This class of nano bone substitutes has shown great promise in a plethora of *in vitro* studies as well as in non-load bearing *in vivo* models. More recently, increasingly sophisticated bio-hybrid composites were developed, which appear able to incorporate multi-substituted biomimetic apatite phases. For example, a Mg-doped apatite/type I collagen nanocomposite was shown to closely resemble the structure and composition of the human trabecular bone niche, significantly improving the osteogenic differentiation of human mesenchymal stem cells (MSCs) *in vitro* and bone regeneration in both ectopic (Minardi et al., 2015a) and orthotopic large animal models (Minardi et al., 2019). Using this biologically inspired synthesis method, some researchers are currently working to develop bio-inspired hybrid nanocomposites with enhanced osteogenic features endowed with magnetic properties (D’Amora et al., 2016; Tampieri et al., 2016). Their potential for bone regeneration will be discussed further in section “Magnetically Responsive Materials” of this review.

### Nanostructured Bio-Glasses

Bioactive glasses are mainly comprised of calcium oxide, silicate, borate, and phosphorous (Hench et al., 2014). By varying the relative amounts of these components, different bioactive glasses can be manufactured and, over the past three decades, many variants have been proposed for bone regenerative applications (van Vugt et al., 2017). Several are available clinically (Jones et al., 2016), and have demonstrated biocompatibility, osteoconductivity, and biodegradability (Kong et al., 2018).

Bioglasses can be prepared by melt–quench or sol–gel process (Vichery and Nedelec, 2016). While the first generations of bioglasses were solid or macroporous, the latest nanostructured versions, synthesized through the sol–gel approach, have unique nanostructural features, including improved nanotextural properties, highly ordered structure, and controlled pore size and pore interconnectivity (Islam et al., 2017; Mancuso et al., 2017). Such nano-features greatly enhance osseointegration compared to first generation bulk bioglasses. The graft-bone integration begins with the solubilization of surface ions resulting in a silica gel layer. A nanostructured calcium phosphate phase (i.e., hydroxyapatite) starts to nucleate on this layer, activating local osteoblasts to form new bone (Ducheyne, 2015). This mechanism contributes to the nano-bioglass degradation, while promoting bone formation. Even their degradation depends on their composition and nanostructure and can be tailored from days to months; for example, borate-based bioglasses have been shown to degrade much faster than silicate varieties (Balasubramanian et al., 2018; Furlan et al., 2018). Recent studies showed that increasing the surface area and porosity of nanostructured bioglasses can greatly accelerate their biodegradation, as well as biointegration (Kong et al., 2018).

The ability to release bioactive ions during degradation is one of the most important features of these bioglasses (Mouriño et al., 2019). For instance, it is known that the early vascularization of biomaterials plays an essential role in bone regeneration (Almubarak et al., 2016). Toward this end, numerous nanoparticles and mesoporous bioactive glasses have been specifically developed to enhance not only osteogenesis but also early angiogenesis through the release of pro-angiogenic ions (Kim J.-J. et al., 2017). Namely, strontium-doped bioglass nanoparticles have shown to increase both osteoblast activity (Fiorilli et al., 2018; Leite et al., 2018; Zhao et al., 2018), as well as induce osteoblasts to secrete angiogenesis-associated cytokines for early vascularization, ultimately resulting in improved bone repair (Zarins et al., 2016; Zhao et al., 2018). Similarly, bioglasses releasing copper or cobalt ions have also been proposed, due to their angiogenic properties (Bari et al., 2017; Weng et al., 2017; Kargozar et al., 2018; Zheng et al., 2018). Silver- (Kaya et al., 2018) or manganese-doped nanobioglasses (Nawaz et al., 2018) have instead been developed to deliver antimicrobial activity, and to aid in the healing process by preventing infections.

Due to their highly ordered mesopores and surface area, nanobioglasses can also be excellent delivery vehicles for bioactive molecules (e.g., drugs and proteins) to further boost bone repair (Baino et al., 2017; Wang et al., 2019; Lalzawmliana et al., 2020). For example, Lee J.-H. et al. (2017) reported a significant enhancement in osteoblast activity, secretion of ECM molecules and calcification through the controlled release of phenamil (a drug known as a potent BMP signaling activator) and strontium ions from mesoporous bioglass nanoparticles. In recent proof-of-concept *in vivo* studies, others have demonstrated how mesoporous nanobioglasses can also be an ideal delivery system for growth factors, such as IGF (Lalzawmliana et al., 2019) or FGF (Kang et al., 2015), with significantly imporved regenerative outcomes in preclinical animal models.

## Mimicking the Electrical Environment of Bone: Nanomaterials Harnessing Piezoelectricity, Conductivity and Magnetism

The field of bone mechanobiology has vastly improved since the advent of nanotechnology, expanding our fundamental knowledge of how mechanical forces regulate the process of bone homeostasis and remodeling (Chen et al., 2010). Although the origin remains a topic of debate, mechanical stress-generated electric potentials are known to be important in modulating cellular behavior to control growth and the remodeling process (Perez et al., 2015; Ribeiro et al., 2015b; Zhang et al., 2016). In addition to stress-generated potentials, electric fields present endogenously in living tissues, as well as electrical stimulation applied externally have also been shown to influence cell behavior and promote tissue growth (McCaig et al., 2005; Balint et al., 2013; Kang et al., 2014; Reid and Zhao, 2014; Funk, 2015). Accordingly, manipulation of the electrical environment has emerged as a promising strategy to enhance bone regeneration, with nanotechnological approaches offering tremendous potential for achieving this aim. Collectively, the nanomaterials recapitulating or leveraging the physical stimuli naturally present in the bone can be categorized as (i) piezoelectric, (ii) electrically conductive, and (iii) magnetic materials and are briefly summarized in Table 2. The impact of nanotechnology on their development and rational design is reviewed below.

**TABLE 2:** Advantages and disadvantages of nanostructured materials harnessing physical stimuli for bone regeneration.

	**Advantages**	**Disadvantages**	**References**
**Nanostructured piezoelectic materials**			
Piezoelectric Ceramics (e.g., BT, BN, ZnO)	Robust piezoelectric characteristics Desirable osteoinductive potential	Potential for cytotoxicity	Maeder et al., 2004; Boccaccini and Blaker, 2005; Opoku et al., 2015; Panda and Sahoo, 2015; Rocca et al., 2015; Fernandes et al., 2016; Li X. et al., 2016; Zhang et al., 2016; Bramhill et al., 2017; Damaraju et al., 2017; Ribeiro et al., 2017; Tajbakhsh and Hajiali, 2017; Ehterami et al., 2018; Kao et al., 2019
Piezoelectric Polymers (e.g., PVDF and its copolymers, PLLA, PHBV)	Biocompatibility and non-toxicity Manufacturing flexibility High strength and impact resistance	Unfavorable biodegradability	
Piezoelectric Polymer-Ceramic Composites	Ability to tailor and enhance several properties of the composite construct: mechanical properties, piezoelectric coefficient, biodegradability, bioactivity	Lack of data regarding the piezoelectric properties of certain composite materials	
**Nanostructured electrically conductive materials**			
Conductive Nanomaterials (non-polymeric, e.g., graphene, gold nanoparticles)	Excellent mechanical properties High electrical conductivity ensuring reliable delivery of bioelectric signals	Non-degradability Questions/concerns regarding biocompatibility and long-term safety	Kim S. et al., 2011; Otero et al., 2012; Bitounis et al., 2013; Liu et al., 2013; Nurunnabi et al., 2015; Sridhar et al., 2015; Assaf et al., 2017; Kim J.W. et al., 2017; Silva et al., 2017; Wang et al., 2017; Zhou et al., 2017; Chan et al., 2018; Cheng et al., 2018; Lalegul-Ulker et al., 2018
Conductive Nanopolymers (e.g., polyheterocycle family of conductive polymers)	Improved biocompatibility and biodegradability Manufacturing flexibility	Unfavorable mechanical properties and processability Relative lack of animal studies evaluating *in vivo* performance	
**Nanostructured Magnetically Responsive Materials**			
Magnetic Nanoparticles (MNPs) and Magnetoelectric Composites	Superparamagnetic properties Ability to deliver cues via remote (external) stimulation	Uncertain biocompatibility and long-term safety	Pisanic et al., 2007; Häfeli et al., 2009; Huang et al., 2009; Bock et al., 2010; Wu et al., 2010b; Wu Y. et al., 2010; Wei et al., 2011; Zhu et al., 2011; Panseri et al., 2012; Tampieri et al., 2012; Alarifi et al., 2014; Singh R.K. et al., 2014; Shen et al., 2015; Li X. et al., 2016; Ribeiro et al., 2016; Yun et al., 2016

### Piezoelectric Materials

Piezoelectricity is observed when a mechanical deformation causes the formation of a net dipole moment and subsequent polarization of the material (Tichý, 2010). Bone is a piezoelectric nanostructured material, and this property was invoked as a potential mechanism by which cells could detect and respond to mechanical stress (Fukada and Yasuda, 1957). The role for piezoelectricity in bone remodeling continues to be debated, and there has been renewed appeal for its physiologic importance in the process of bone mechanosensation (Halperin et al., 2004; Noris-Suarez et al., 2007). As such, with the emergence of nanotechnological approaches there has been a rapid increase in the number of publications on piezoelectric materials for bone regeneration (Tandon et al., 2018). They can be thought of as sensitive mechano-electrical transducers, and as such, they are typically applied to the implantation areas which are exposed to mechanical loads (Zhang K. et al., 2018; Chorsi et al., 2019). A number of different piezoelectric materials have been investigated for bone regeneration applications, which are briefly reviewed here.

#### Inorganic Piezoelectric Materials: Piezoelectric Ceramics

Nanopiezoceramic materials investigated for bone regeneration applications include barium titanate (BT), boron nitride (BN), and zinc oxide (ZnO). While these materials possess a high piezoelectric coefficient, some of them display lower biocompatibility at high doses, which can represent a major limitation for their use in tissue engineering applications (Maeder et al., 2004; Opoku et al., 2015; Panda and Sahoo, 2015; Kao et al., 2019). Nevertheless, each of these piezoceramics has shown osteoinductive capabilities *in vitro*, supporting their use in the development of bone regenerative biomaterials, where they are often incorporated in a variety of ways into 3D scaffolds in order to impart piezoelectric characteristics to augment bone formation. For example, BT nanoparticles have been shown to enhance the osteogenic differentiation of MSCs, and osteoblastic cells demonstrated superior adhesion, proliferation, and migration into the pores of scaffolds comprised of BT, while BN nanotubes (BNNTs) demonstrate high protein adsorption ability and promotion of enhanced MSC attachment, proliferation, and osteogenic differentiation (Rocca et al., 2015; Li X. et al., 2016; Tajbakhsh and Hajiali, 2017; Ehterami et al., 2018). Finally, the incorporation of ZnO nanoparticles has proven capable of enhancing both the bioactivity and even the mechanical properties of such composite materials (Shalumon et al., 2011; Feng et al., 2014; Kao et al., 2019).

#### Organic Piezoelectric Materials: Piezoelectric Polymers

Piezoelectric polymers have also received increasing attention for bone regeneration applications in recent years (Tichý, 2010). Typically fabricated either as films, rods, or tubes/fibers (Ribeiro et al., 2015b), they exhibit sound mechanical properties, including superior strength and impact resistance when compared to inorganic materials. Biocompatibility, piezoelectric activity, and significant osteogenic capacity have also been demonstrated both *in vitro* and *in vivo* (Zhang et al., 2016; Damaraju et al., 2017; Ribeiro et al., 2017; Kao et al., 2019). Among these, PVDF [poly(vinylidene fluoride)] and its copolymers, PLLA, and PHBV (poly-3-hydroxybutyrate-3-hydroxy valerate) are the most studied.

Poly(vinylidene fluoride) and its copolymers can provide the necessary electromechanical stimulation for the differentiation of human MSCs into the osteogenic lineage *in vitro* (Damaraju et al., 2013, 2017; Nunes-Pereira et al., 2015; Ribeiro et al., 2015a; Zhang et al., 2016), as well as the capacity to effectively promote bone regeneration *in vivo* in rodent models (Zhang et al., 2016; Ribeiro et al., 2017). In addition to its potential utility as a bone graft substitute, PVDF has also shown promise as a suitable coating for existing implant materials in order to enhance osteogenesis (Zhou Z. et al., 2016). The primary concern with PVDF and its copolymers is the lack of biodegradability, which limits clinical potential. This limitation is being addressed with the development of newer-generation piezoelectric polymer-based materials with tailorable degradation properties. Poly-3-hydroxybutyrate-3-hydroxy valerate and PLLA, both of which are biodegradable, have emerged as promising candidates (Duan et al., 2011), demonstrating osteogenic capacity both *in vitro* and *in vivo* (Ikada et al., 1996; Sultana and Wang, 2012; Wang et al., 2013). PLLA has also been explored for bone regeneration utility beyond its use as a bone graft substitute. Due to its biodegradability, non-toxicity, and advantageous mechanical properties, PLLA is an attractive material for clinical application in the fabrication of biodegradable fixation devices such as screws, pins, and suture anchors, where a bioresorbable implant is desirable to avoid the risk of complicating revision surgery or the requirement for an additional procedure for implant removal (Bucholz et al., 1994; Barber et al., 1995; Prokop et al., 2005; Gkiokas et al., 2012).

#### Piezoelectric Polymer – Ceramic Composite Materials

Piezoelectric polymers and ceramics have also been used in combination to fabricate a variety of composite materials (Boccaccini and Blaker, 2005; Bramhill et al., 2017). Polymer matrix composites harness the manufacturing flexibility afforded by polymers and the substantial piezoelectric properties of otherwise brittle ceramics to produce complex forms ideally suited to support bone formation, including porous scaffolds (Zhang et al., 2014; Liu et al., 2016), layered structures (Dubey et al., 2015), nanoparticles (Marino et al., 2015, 2017; Niskanen et al., 2016), and dense disks (Dubey et al., 2013). Of the polymer matrix composites, PLLA-based composites have been used most extensively in the field of bone regeneration (Fernandes et al., 2016; Tajbakhsh and Hajiali, 2017). Composite membranes incorporating PVDF-TrFE and BT have also been found to support bone formation in several investigations (Gimenes et al., 2004; Scalize et al., 2016; Zhang et al., 2016), suggesting significant potential for clinical application owing to the improved osteogenic capability demonstrated *in vitro* and *in vivo* in rodent bone defect models. Of the ceramic matrix composites, HA/BT-based materials are the most studied, with a number of studies demonstrating the osteoinductive capability of such composites (Jianqing et al., 1997; Baxter et al., 2009; Dubey et al., 2014; Jiao et al., 2017; Ehterami et al., 2018).

The emergence of piezoelectric materials and their rapidly increasing usage has motivated investigators to adopt new and innovative approaches to create biomaterials with desirable properties. Techniques which are gaining interest include 3D printing (Kim et al., 2014; Schult et al., 2016; Bodkhe et al., 2017), fabrication of piezoelectric nanofibers using solution blow spinning (Bolbasov et al., 2014, 2016; Daristotle et al., 2016), and the development of systems capable of applying controlled mechanical stimulation to piezoelectric scaffolds (Trumbull et al., 2016; Zhou X. et al., 2016).

A lack of quantitative data on the piezoelectric coefficient of many composite materials is a limitation to this newly emerging class of materials. However, although this area of research remains in its relative infancy, nanopiezoelectric materials show tremendous promise for bone regeneration.

### Electrically Conductive Materials

In cases when the patient is immobilized, whether due to a fracture or other health condition, or in a non-load bearing healing setting, the natural mechanical stimulus does not occur and the effectiveness of piezoelectric materials is subsequently diminished (Mehta et al., 2012). Such limitations call for the development of new approaches capable of delivering electrical cues via alternative means, either by remote stimulation or through innovative nanomaterials activated by micromotion. Electrically conductive materials provide such an innovative tool, serving as the substrate through which external electrical stimulation is converted into bioelectric signals and delivered to the site (Chen et al., 2019).

Electrical stimulation therapy has occasionally been attempted as a supplement to promote bone healing in the case of fractures and spinal arthrodesis, although with arguable success, for decades (Gan and Glazer, 2006; Goldstein et al., 2010; Einhorn and Gerstenfeld, 2015). Researchers have more recently begun to explore conductive materials capable of propagating electrical signals to the site of repair in order to accelerate bone regeneration. Unlike piezoelectric materials, these require an externally applied power source to produce electrical signals. On one hand, this approach requires optimization of a number of different parameters including the frequency, amplitude, duration, and nature (alternating/direct) of the signal which may complicate assessment of efficacy (Dubey et al., 2011). On the other hand, it affords a great degree of control over the stimulus which cannot be achieved with the use of piezoelectric materials, allowing the functionality of the material to be tailored to its specific application.

One method for producing electroactive biomaterials capable of serving as conduits for the delivery of external electrical stimulation to cells involves the use of a polymer matrix incorporating conductive nanomaterials such as graphene (Assaf et al., 2017), carbon nanofibers (Whulanza et al., 2013), or metallic particles (e.g., gold nanoparticles) (Sridhar et al., 2015). Of these, graphene family materials have been found to possess excellent mechanical and conductive properties (Kim S. et al., 2011; Bitounis et al., 2013; Kim J.W. et al., 2017), support proliferation (Kalbacova et al., 2010) and osteogenic differentiation of MSCs (Nayak et al., 2011; Bressan et al., 2014), yield high degrees of mineralization (Lee et al., 2011; Xie et al., 2015), and even exert antimicrobial action (Pang et al., 2017). A number of graphene-based materials have been developed in the form of scaffolds, scaffold reinforcement materials, and surface coatings for existing materials, with demonstrated capacity to promote and enhance new bone formation *in vivo* (Silva et al., 2017; Wang et al., 2017; Zhou et al., 2017). Significant limitations to graphene and other similar electroactive materials include their non-degradability and uncertain biocompatibility, as well as questions regarding their long-term safety (Nurunnabi et al., 2015; Cheng et al., 2018).

To address these limitations, other methods of obtaining electroactive biomaterials which utilize intrinsically conductive polymers (CPs) have been explored. Such an approach offers the advantages of improved biocompatibility and biodegradability, in addition to manufacturing flexibility allowing incorporation of other components (Lalegul-Ulker et al., 2018). Among several CPs in use, the polyheterocycle family, including polypyrrole (PPy), polyaniline (PANI), and polythiophene (PTh) and its derivative poly(3,4-ethylenedioxythiophene) (PEDOT), are the most extensively studied for bone regeneration applications (Otero et al., 2012). These materials exhibit desirable electrical conductivity sufficient to promote cell proliferation and osteogenic differentiation (Liu et al., 2013), but are limited by inherently poor mechanical properties and processability (Chan et al., 2018), prompting the development of conductive polymeric composites. For example, CPs can be blended with various other natural and/or synthetic non-CPs to fine-tune degradation and mechanical properties (Kaur et al., 2015). Conductive copolymers incorporating other electroactive polymeric components provide for further enhancement of biocompatibility, biodegradability, and electroactivity (Cui et al., 2012). Conductive polymer-based conducting nanofibers, conducting hydrogels, and 3D conductive composite scaffolds are additional examples of electroactive biomaterials being explored for bone regeneration applications (Sajesh et al., 2013; Li L. et al., 2016; Guex et al., 2017; Chen et al., 2018). While numerous investigations have generated exciting results supporting the osteogenic capabilities of conducting polymers and their composites *in vitro*, there remains a need for more animal studies to validate the performance of this promising family of electroactive biomaterials.

### Magnetically Responsive Materials

Magnetic stimulation therapy, like electrical stimulation therapy, has been used clinically for a number of years (Assiotis et al., 2012). While the underlying mechanisms of action are unclear, *in vitro* studies suggest that pulsed and static magnetic fields are capable of enhancing osteoblast differentiation (Jansen et al., 2010; Wang et al., 2014; Marędziak et al., 2016), and animal studies have shown promise for promoting bony healing and integration into graft materials (Fredericks et al., 2000; Puricelli et al., 2006).

When describing the magnetic behavior of a material, ferro- and ferrimagnetism refer to a material’s ability to be magnetized by an external magnetic field and remain magnetized upon its removal. Paramagnetism, on the other hand, is defined by a material’s lack of retained magnetism upon removal of the external magnetic field, a desirable property in tissue engineering applications, as aggregation of the material’s magnetic particles *in vivo* could lead to local toxicity (Balavijayalakshmi et al., 2014). Of particular interest are magnetic nanoparticles (MNPs) owing to their special superparamagnetic properties. Superparamagnetic behavior, exhibited by small ferro- or ferrimagnetic nanoparticles, do not retain magnetism in the absence of external magnetic fields; however, their magnetic susceptibility is much greater than that of standard paramagnetic materials, permitting precise magnetic control and functionalization for a given application (Reddy et al., 2012). Among MNPs, iron oxide nanoparticles, typically maghemite (Fe_2_O_3_) or magnetite (Fe_3_O_4_), have been the most commonly used (Liu et al., 2016), as they have demonstrated osteoinductive capacity *in vitro*, even in the absence of external magnetic stimulation (Huang et al., 2009; Bock et al., 2010; Wei et al., 2011). Thus, MNPs have been incorporated into conventional bioceramic or polymeric scaffolds, adding intrinsic magnetic properties capable of enhancing osteogenic potential. Results from *in vivo* studies suggest that the magnetic field resulting from the presence of incorporated MNPs, albeit small, can indeed drive the formation of new bone, even without external magnetic stimulation. Wu and colleagues incorporated iron oxide MNPs into a CaP bioceramic scaffold and found this material capable of enhancing osteogenesis in a rodent model of ectopic bone formation (Wu Y. et al., 2010), while Singh and associates produced a PCL biopolymeric nanofibrous scaffold incorporating iron oxide MNPs, which demonstrated the ability to enhance bone formation in a rodent segmental bone defect model (Singh R.K. et al., 2014).

MNP incorporation provides further functionality by rendering the biomaterial magnetically responsive, permitting the use of controlled external magnetic field stimulation to potentially regulate and direct cellular behavior toward osteogenesis and even angiogenesis (Sapir et al., 2012). Yun et al. (2016) studied the effects of external magnetic stimulation applied to magnetic PCL/MNP scaffolds on osteoblast differentiation and bone formation and found that external stimulation not only promoted *in vitro* osteoblastic differentiation, but also significantly enhanced new bone formation, compared to the magnetic scaffold alone, in mouse calvarium defects.

New and innovative methods in this arena continue to emerge. In a combined approach, magnetoelectric composite materials bridge the magnetic and piezoelectric properties of bone to produce a potentially synergistic regenerative effect. Such materials respond to magnetic stimulation with mechanical deformation (due to the *magnetostriction* of one component, that is the change in shape occurring during magnetization), resulting in electrical polarization (due to the piezoelectric behavior of the other component). Thus, bioelectrical cues can be delivered to a desired cellular environment with precise remote control (Ribeiro et al., 2016).

Since their introduction, concerns regarding the cytotoxic effects of iron oxides have justifiably arisen, with a documented relationship between their clinical use and the outbreak of acute adverse events, such as nephrogenic systemic fibrosis, formation of apoptotic bodies, inflammation, and other toxic effects (Pisanic et al., 2007; Häfeli et al., 2009; Wu et al., 2010b; Zhu et al., 2011; Alarifi et al., 2014; Shen et al., 2015). This has provoked efforts to produce magnetic biomaterials with improved biological features, such as doping well-known biocompatible nanomaterials with a magnetic phase to replace magnetite and the other iron oxides. Recently, Tampieri and colleagues reported fabrication of biocompatible FeHA nanoparticles with a superparamagnetic-like phase by doping HA with iron (Fe^2+^/Fe^3+^) ions (Tampieri et al., 2012). *In vitro* studies showed that these FeHA nanoparticles were capable of enhancing cell proliferation to a greater degree than HA particles alone, without reducing cell viability. Furthermore, the *in vivo* biocompatibility of FeHA was demonstrated in a pilot animal study of a rabbit critical bone defect (Panseri et al., 2012). While approaches to bone regeneration based on magnetic stimulation and magnetically responsive biomaterials are in the early stages of development, the results to date suggest promise for such strategies in bone regeneration applications going forward.

## Materials Mimicking Bone Architecture: 3D Printed and Biomorphic Ceramics

Native bone displays structural features with levels of organization spanning several orders of magnitude (nm to cm scale) (Chen et al., 2008). This multiscale hierarchical structure, as well as the interactions between its organic and mineral components at the molecular level, contribute significantly to biological and mechanical properties of bone (Gupta et al., 2005). Thus, utilization of these features to guide the hierarchical design of biomaterials represents a potential strategy to promote bone regeneration. This section focuses on nanostructured scaffold materials designed to recapitulate native nanocues by providing mimicry of the structural features of the natural bone matrix.

### Architectural Considerations

For bone tissue engineering applications, a scaffold should possess appropriate structural and mechanical properties to sustain physiological loads in order to preserve weight-bearing function, while also possessing intrinsic biocompatibility in order to facilitate favorable biomaterial-native bone interactions, which serve to enhance tissue regeneration and implant integration (Ikeda et al., 2009). Many early bone tissue engineering designs sought to accomplish this goal through synthetic structures which imparted bulk properties to the constructs, such as adequate mechanical strength and sufficient transport properties for cell infiltration and tissue organization (Christenson et al., 2007). These designs, although successful in replicating many of the macroscopic properties of native bone, often failed prior to full healing (Burdick and Anseth, 2002; Murugan and Ramakrishna, 2005). A key factor identified in these failures was inadequate tissue regeneration around the material shortly after implantation, owing to poor interaction of the biomaterial with the host tissue (Christenson et al., 2007). In fact, the process of bone formation is governed by interactions and informational cues derived from structural features spanning multiple length scales from nanoscale to macroscale (Gusic et al., 2014). Nanoscale interactions in particular have been shown to be crucial in controlling cell functions such as proliferation, migration, and adhesion in native tissues (Benoit and Anseth, 2005). Indeed, all living systems are governed by molecular behavior at nanometer scales (Zhang et al., 2012). As in other tissues, the cellular organization and corresponding tissue properties of bone are highly dependent on the nanostructural features of the ECM, since cells are predisposed to interact with nanostructured surfaces (Kaplan et al., 1994; Taton, 2001; Liu et al., 2006). This may help to explain why early generation tissue substitutes—produced through macro- and microfabrication techniques that were unable to recreate sophisticated structures that mimic the subtleties of the ECM—showed suboptimal performance. Recent paradigm shifts to nanoscience-enabled techniques have resulted in the emergence of novel nanotechnological approaches that enable more precise recapitulation of the architectural features of native bone, offering greater potential for modulating cellular behavior and enhancing bone regeneration (Webster et al., 2000; Murphy et al., 2010; Saiz et al., 2013; Tang et al., 2016).

Native bone is characterized by unique topological features derived from its micro- and nanostructured surfaces and interfaces, which are crucial to its function and growth and therefore promising targets for biomimicry (Nadeem et al., 2015). Nanotechnology offers new opportunities to capitalize on the structure-function relationships in bone by replicating a number of these integral features. By providing the substrate upon which cells attach and proliferate, surface topography can modulate cellular behavior and function (Boyan et al., 2002). Native bone is composed of collagen fibrils with rod or needle-like HA deposits scattered across their surface. These deposits produce surface roughness which has been shown to promote both adhesion of osteoblasts as well as differentiation of MSCs to the osteogenic lineage (Nadeem et al., 2015). Based on this, researchers have developed approaches to introduce surface roughness onto scaffold materials in order to more effectively mimic the mineralized interface encountered by cells adhering to native bone ECM (Henkel et al., 2013). Farshid and colleagues (Farshid et al., 2017), for instance, introduced microscale surface roughness onto polymeric scaffolds through the incorporation of boron nitride nanotubes and nanoplatelets, which resulted in greater collagen deposition and cell attachment by pre-osteoblasts *in vitro*. In another approach, Shakir et al. (2018) utilized nano-HA to enhance the surface roughness of a resin-based chitosan scaffold, which they found capable of promoting superior bone regeneration *in vivo* in a rat calvarium defect model.

Given that the HA deposits producing surface roughness in native bone have dimensions in the nanoscale, fabrication of surfaces with nanostructured topography can prove even more beneficial to inducing osteogenesis than simply producing roughness at the microscale (Lim et al., 2005). Indeed, Lim and colleagues (Lim et al., 2005) generated nanoscale surface roughness by introducing “nanoislands” of varying size to a polymeric substrate and investigated their effects on osteoblastic cell behavior. They found that a smaller island height produced greater cell adhesion and spreading as well as increased alkaline phosphatase activity, demonstrating the advantages of down-scaling the dimensions of topographical features. Other surface nanotopographies, such nanogrooves and nanopits, have also been shown to enhance osteoblast differentiation and osteogenic cell function in several studies (Dalby et al., 2007; Liu et al., 2014; Gong, 2015; Xu et al., 2017).

In addition to surface topography, cell and ECM alignment within the native bone represents a structural feature integral to its growth and function, and is thus a promising target for biomimicry (Takano et al., 1999). The anisotropic characteristics of bone tissue—a result of its unique adaptive response to external forces—is due to the longitudinal alignment of its collagen fibers, and there is evidence that MSCs more readily differentiate to an osteogenic phenotype when confined into such an alignment (Ber et al., 2005; Li et al., 2007). This phenomenon is thought to be mediated by contact guidance mechanisms whereby instructive physical cues, generated through the local interactions which occur in specific cellular orientations and alignment, act to regulate cell morphology and function (Boyan, 1996; Badami et al., 2006). Tissue engineering strategies which are capable of exploiting these mechanisms may therefore allow cell fate to be precisely directed for its intended application. For the purposes of bone tissue engineering, simulation of the alignment found in the native bone may potentially promote bone regeneration by driving stem cells toward an osteogenic lineage and enhancing their functions through the recapitulation of the native cues (Ber et al., 2005; Nadeem et al., 2015).

To achieve the desired alignment, one approach involves the creation of micron and/or nanoscale grooves on the substrate material, which allows cells to grow and spontaneously elongate along the direction of groove alignment (Perizzolo et al., 2001; Zhu et al., 2005; Badami et al., 2006). Nadeem et al. (2015) utilized such an approach through the introduction of integrated surface micropatterns to their 3D CaP/gelatin biomaterials, producing cell-instructive scaffolds which were osteoinductive *in vitro* and promoted greater bone formation and osseointegration *in vivo* in a rabbit radial segmental defect model. A more direct approach toward biomimicry is to simply replicate the aligned fibers seen in the native collagenous architecture of bone. Innovative techniques utilizing aligned nanofibers created, for example, by electrospinning, have made it possible to accomplish this form of biomimicry with extraordinary precision (Jose et al., 2009; Anjaneyulu et al., 2017). By replicating the morphological and chemical structure of the natural ECM at the nanoscale level, nanofibrous scaffold materials offer greater potential to modulate cellular function and guide cell growth (Murugan and Ramakrishna, 2005; Leung and Ko, 2011; Pas̨cu et al., 2013). Additionally, such materials provide increased surface area-to-volume ratios and porosity, thereby enhancing osteoconductivity, as well as desirable biocompatibility, biodegradability, and mechanical strength (Haider et al., 2018).

### 3D Printing

The internal porosity of native bone is yet another important structural feature which bone regenerative engineering approaches have targeted for biomimicry. The presence of an interconnected, 3D, porous architecture is a critical requirement for any bone tissue engineering strategy in order to allow for cell migration and the transport of nutrients and waste (Lee et al., 2014). Nanofibrous scaffolds prove especially advantageous in this regard, as the small fiber diameter creates a highly porous matrix enabling effective cell migration and proliferation throughout the scaffold (Rezwan et al., 2006; Zhang et al., 2008; Xu et al., 2017). In addition to overall porosity, average pore size is another significant consideration. Although the optimal pore size to promote bone regeneration within engineered scaffolds has not been definitively established, in general, smaller pore sizes will promote initial cell adhesion due to higher substrate surface area, while larger pores will enable greater cellular infiltration from surrounding tissue, a critical requirement for vascular ingrowth and subsequent tissue maintenance (Kenar et al., 2006; Murphy et al., 2010; Cox et al., 2015). While nanofibrous scaffolds provide a high overall porosity, nanofibers created by electrospinning tend to produce constructs with reduced average pore size compared to larger fiber scaffolds, resulting in decreased cell penetration depth (Badami et al., 2006). The need for more precise control of porosity and pore size within scaffold materials has prompted the implementation of novel 3D printing systems which may offer such capabilities. 3D printing technologies such as fused deposition modeling, stereolithography, and selective laser sintering have enabled the production of scaffolds with greater spatial resolution and fidelity than traditional fabrication methods, while also offering the ability to introduce precise pore gradients which more effectively mimic the physical cues for growth found in native bone tissue (Bracaglia et al., 2017; Alehosseini et al., 2018; Malikmammadov et al., 2018; Babilotte et al., 2019). While 3D printing approaches to the design of scaffolds for bone tissue engineering are quite new and still being explored for their utility, they also offer strong potential for the 3D patterning of surface roughness and other key physical features, providing even further recapitulation of the native cues present in bone (Murphy and Atala, 2014).

Mimicking the architecture of native bone is an essential component of material design for bone regeneration applications. These materials must provide an environment suitable for cellular recruitment, adhesion, proliferation, and pro-osteogenic differentiation. There is an abundance of technologies that provide tight control over topography, porosity, and mechanical properties of various materials that have proven useful for bone regeneration. Providing a suitable environment for osteogenesis is a crucial aspect of material design for bone regeneration, but it is not the only consideration. These materials must also be durable, biocompatible, and capable of integrating with surrounding tissues, among other properties, to be relevant for clinical applications.

### Scaffolds Synthesized Through Biomorphic Transformation

Long bone and critical-sized defects caused by trauma, non-union, or tumors represent a difficult clinical challenge in need of more reliable solutions (Berner et al., 2012; Roffi et al., 2017). Most currently available synthetic scaffolds have not proven capable of providing the necessary osteo- and vascular conductivity within the innermost portions of the scaffold. This could be attributed to a disorganized and tortuous porosity impeding cell penetration into the scaffold and subsequent tissue development; sufficient mechanical strength to promote integration with host tissues can also be a challenge (Mastrogiacomo et al., 2006).

In the attempt to overcome these limitations, “biomorphic transformations” have been developed. These synthetic approaches consist of a series of steps involving pyrolysis and complex chemical reactions (mainly liquid or gas infiltration processes), allowing for the chemical transformation of natural substrates into ceramic scaffolds, while preserving their original fine architecture from the nano up to the macro scale. Among natural templates, one is particularly advantageous as a solution to long bone defect healing—wood. Wood presents a unique hierarchical architecture on a cellular micro and nano-structure scale (Fratzl and Weinkamer, 2007). The pattern of fiber bundles and channel-like porous areas of selected types of wood (e.g., rattan) is surprisingly similar to that found in long bone (Tampieri et al., 2009). There have been a few remarkable attempts to utilize wood as a scaffold for the synthesis of biomimetic hierarchically organized load bearing scaffolds for long bone repair. In 2009, a biomimetic HA bone scaffold from natural wood with highly organized multiscale porosity was first proposed (Tampieri et al., 2009). The resulting material was a porous nanostructured apatite scaffold with a hierarchical structure, representing an inorganic substitute for bone graft that allowed for cellular invasion while providing space for vascularization (Tampieri et al., 2009). Recently, they used a similar approach of bio-ceramization of a wood template to prepare a hollow cylindrical ceramic scaffold to resemble cortical bone, and filled it with a spongy HA/collagen bio-hybrid scaffold to resemble spongy bone. They assessed the osteoconductivity of the construct in a sheep critical size load bearing model (2 cm metatarsus defect), finding significant osteointegration at the bone/scaffold interface (Filardo et al., 2014). Using the same large animal model, in a follow-up study, they increased the diameter of the lumen of the external cortical-like biomorphic scaffold (Filardo et al., 2019). Osteointegration was observed in all samples, but the group with the largest internal diameter (11 mm) showed the best results in terms of bone-to-implant contact and new bone growth inside the scaffold. Additionally, the investigators posited that scaffold degradation in the cortical area—which induced osteointegration and new bone formation—is possible evidence of activation of load-induced biochemical signaling within the bone healing cascade.

Bigoni et al. (2020) reported that the mechanical properties of these biomorphic HA scaffolds have superior mechanical properties (higher strength, stiffness, and toughness at low density) when compared to usual porous ceramics obtained through sintering; probably due to the unique hierarchically organized multiscale resolution down to the nano-scale, which is not yet present in common ceramics.

While there is much potential for wood-based scaffolds and biomorphic transformation, certain drawbacks exist in comparison with other approaches. For instance, the process of biomorphic synthesis requires complex and strict control of reaction kinetics to avoid deformations and structural defects and to maintain the multiscale porosity (i.e., down to the nanoporosity) (Tampieri et al., 2018). Further, it relies on gas-solid reactions that are strongly affected by different phenomena relating to adsorption of the gaseous reactant by the solid, kinetics of nucleation and growth of synthesized inorganic phase at the surface, and the penetration of the gaseous reactant within the innermost portion of the structure (Szekely, 2012). This control is vital when fabricating larger pieces, since diffusive phenomena affect the rate of phase transformation (Bigoni et al., 2020). Without strict control of this process, the advantages of wood as a template cannot be capitalized upon.

## Mimicking Bone’s Biochemical Niche: Delivery of Bioactive Molecules

**FIGURE 3:**
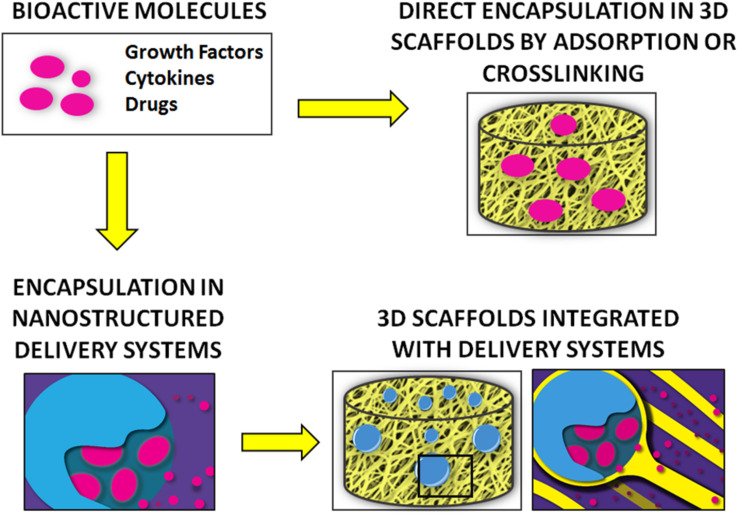
Summary of the main strategies for the delivery of bioactive molecules for bone regeneration.

A variety of bioactive molecules compose the biochemical milieu of bone (Minardi et al., 2020). Several strategies have been proposed to deliver biochemical cues (e.g., growth factors, cytokines) to recapitulate this environment and enhance bone regeneration, as summarized in Figure 3. Initial attempts consisted of the direct adsorption or crosslinking of biomolecules to implants, which resulted in suboptimal outcomes, mostly due to burst release and molecule denaturation (Fan et al., 2012).

Delivery systems offer more effective and precise control over release (Minardi et al., 2014). Among delivery systems, nanostructured varieties have proven superior, as they can be finely tuned to provide a higher yield of loading and sustained release over time, while allowing for complex temporally controlled release kinetics (Minardi et al., 2016b). The most common nanostructured delivery systems developed for bone regeneration are reviewed below and summarized in Table 3.

**TABLE 3:** Main types of nanostructured delivery systems used in bone regeneration, with their respective advantages and disadvantages.

	**Advantages**	**Disadvantages**	**References**
**Inorganic nanostructured delivery systems**			
Ceramics (e.g., HA, TCP)	Intrinsic osteoconductivity Surface functionalization Widely available	Unfavorable biodegradability profile Low yield of payload loading	Matsumoto et al., 2004; Dong et al., 2007; Habraken et al., 2007; LeGeros, 2008; Yuan et al., 2010; Xie et al., 2010; Bose and Tarafder, 2012; Jeon et al., 2012; Fielding and Bose, 2013; Fan et al., 2014; Wen et al., 2017
Metallic or metalloid oxides (e.g., silica)	Tailorable mesoporous structure Surface functionalization with and/or encapsulation of bioactive molecules Modifiable architecture and topography Optimization of cell adhesion and proliferation	Cytotoxicity at certain particle sizes and/or concentrations	Oh et al., 2005; Raja et al., 2005; Magrez et al., 2009; Lai et al., 2011; Lallana et al., 2012; Portan et al., 2012; Tang et al., 2012; Setyawati et al., 2013; Shadjou and Hasanzadeh, 2015; Zhou et al., 2015; Cui et al., 2018; Tang et al., 2014; Hu et al., 2012; Huang et al., 2014; Kwon et al., 2017; Liu et al., 2017
**Organic nanostructured delivery systems**			
Synthetic polymers (e.g., PLA, PLGA)	Widely available Overall favorable biocompatibility Many modifiable properties: e.g., L/G ratio, molecular weight. Modifiable with cross-linkers or surface functionalization	Low yield of payload loading Burst release Difficulty in accomplishing sustained release Certain polymers have cytotoxic degradation products	Alcantar et al., 2000; Habraken et al., 2007; Lü et al., 2009; Puppi et al., 2010; Anderson and Shive, 2012; Makadia and Siegel, 2011; Jacob et al., 2018
Natural polymers (e.g., gelatin, chitosan)	Widely available Favorable biocompatibility and biodegradability Biomimetic properties Modifiable with cross-linkers or surface functionalization	Low yield of payload loading Rapid degradation *in vivo* Burst release Difficulty in accomplishing sustained release	Friess, 1998; Aframian et al., 2002; Malafaya et al., 2007; Niu et al., 2009; He et al., 2011; Vo et al., 2012; Farokhi et al., 2014; Amjadian et al., 2016; Cai et al., 2016; Ding et al., 2016; Shen et al., 2016; Jacob et al., 2018; Oliveira et al., 2019
**Composite nanostructured delivery systems**			
Composites	High loading efficiency Highly tunable release kinetics Sustained release Optimization of unique properties of each material	Generally require more complex syntheses	Li et al., 2006; Liu et al., 2009; Niu et al., 2009; Reves et al., 2011; Fan et al., 2012; Singh et al., 2015; Minardi et al., 2015b; Kim B.-S. et al., 2018; Wang et al., 2018; Zhang Q. et al., 2018; Minardi et al., 2020

### Nanostructured Delivery Systems

Osteogenic growth factors, including bone morphogenetic proteins (BMP-2 and BMP-7), or the transforming growth factor-β (TGF-β) family, are known to play a crucial role in cell proliferation, differentiation, and ultimately osteogenesis (Chen et al., 2004). As FDA-approved in 2002, BMP-2 is delivered with an absorbable collagen sponge (ACS) [INFUSE^TM^] for clinical applications (McKay et al., 2007). Although efficacious, supraphysiologic doses of the growth factor are required, which have been associated with a number of adverse side effects (Tannoury and An, 2014). Given these challenges, there is significant research interest in the development of novel delivery systems to provide controlled release of lower doses of BMP-2 and other bioactive molecules important for bone regeneration. Toward this end, a wide array of nano-structured systems capable of delivering bioactive signals and molecules have been proposed.

#### Inorganic Nanostructured Delivery Systems

Utilizing ceramic materials for drug delivery in the field of bone regeneration presents advantages, as these materials themselves have osteoconductive properties (Habraken et al., 2007; LeGeros, 2008; Yuan et al., 2010). Commonly used ceramics include CaPs, such as HA and TCP. In the first generation of HA-based delivery systems, HA was directly adsorbed with bioactive molecules such as BMP-2 (Matsumoto et al., 2004; Dong et al., 2007; Xie et al., 2010), however, side effects associated with their burst release quickly demanded alternative strategies (Xie et al., 2010), such as chemically bonding bioactive molecules to the surface of the ceramic particles, which provides a more controlled and sustained release over time (Fan et al., 2014). The surface of CaP particles can be functionalized to bind a wide array of bioactive molecules for bone regeneration (Bose and Tarafder, 2012). For example, nano-HA particles can be functionalized to bind to and provide sustained release of BMP-2 to stimulate osteogenesis *in vitro* (Jeon et al., 2012).

Metallic or metalloid oxide nanomaterials such as silica (SiO_2_) and titanium oxide (TiO_2_) nanotubes have also been functionalized into nano-structured delivery systems for different bioactive molecules for bone regenerative applications (Lai et al., 2011; Shadjou and Hasanzadeh, 2015; Zhou et al., 2015). Silica-based nanomaterials (e.g., mesoporous silica) have been engineered to provide controlled release of different biomolecules (Shadjou and Hasanzadeh, 2015). These materials are generally biocompatible and can be easily functionalized with a number of different linker molecules (Lallana et al., 2012). Zhou et al. (2015) utilized silica nanoparticles to enable dual-delivery of BMP-2 and dexamethasone, and Cui et al. (2018) have utilized a silica-based nanomaterial delivery system for controlled release of BMP-2-related peptide both *in vitro* and *in vivo*. The tailorable mesoporous structure and the ability to bind a variety of different molecules are notable advantages of these silica-based materials (Tang et al., 2012). Additionally, the architecture and topography of these compounds can be engineered to promote cell adhesion, proliferation, and differentiation—all critical requirements for *in vivo* applications (Tang et al., 2014).

TiO_2_ nanotubes for delivery of drugs and other biomolecules have also been described (Hu et al., 2012; Huang et al., 2014; Kwon et al., 2017). These can be designed to both encapsulate and display the molecule of interest on the material surface (Huang et al., 2014). One group directly functionalized the surface of TiO_2_ nanotubes with BMP-2, which promoted osteogenic differentiation *in vitro* (Lai et al., 2011). In addition to biomolecule delivery, the surface of TiO_2_ nanotubes can be activated and coated with ceramics like CaP or HA (Oh et al., 2005; Raja et al., 2005). However, concerns have arisen regarding the toxicity of TiO_2_-based nanomaterials, with one study suggesting that the strong adherence of osteoblasts to the metallic material may induce apoptosis (Portan et al., 2012; Setyawati et al., 2013; Liu et al., 2017). Dose-dependent cytotoxic effects of TiO_2_ nanofilaments have also been described elsewhere (Magrez et al., 2009).

#### Organic Nanostructured Delivery Systems

Alternatively, polymer-based delivery systems have been fabricated using both synthetic and natural materials (Jacob et al., 2018). Commonly used synthetic polymers include polyethylene glycol (PEG), poly(L-lactic acid) (PLA), PCL, PLGA, and poly(L-lactic acid) fumarate (PLAF). Polyethylene glycol and PLA are comprised of single monomers, while PCL, PLGA, and PLAF are copolymers. There has been extensive use of synthetic polymers as delivery systems for bone regeneration applications, including delivery of BMP-2, dexamethasone, antibiotics, and other pharmacologics (Puppi et al., 2010). Polyethylene glycol, PCL, and PLGA are all biocompatible (Alcantar et al., 2000; Anderson and Shive, 2012), although PLGA is generally favored, because it is FDA-approved and has been demonstrated to be non-inflammatory in various studies (Habraken et al., 2007; Lü et al., 2009; Makadia and Siegel, 2011). Additionally, various properties of PLGA—the L/G ratio, molecular weight, and stereochemistry—can be modified to control the polymer’s properties and degradation rate (Habraken et al., 2007). Polymers such as PLA and PLGA can yield cytotoxic acidic degradation products (Habraken et al., 2007). Therefore, controlled degradation is important for both drug delivery and to minimize toxicity.

Natural polymers used for the controlled release of bioactive molecules to promote bone regeneration include gelatin, chitosan, alginate, collagen, silk fibroin, hyaluronic acid, and fibrin, among others (Jacob et al., 2018). These materials are advantageous given their biocompatibility and biomimetic properties, which result from a close resemblance of native ECM, and are also fully biodegradable (Malafaya et al., 2007; Vo et al., 2012). Natural polymers have been described in systems delivering BMP-2 (Niu et al., 2009; Shen et al., 2016), vascular endothelial growth factor (VEGF; Farokhi et al., 2014), antibiotics (Cai et al., 2016), and immunomodulators (Amjadian et al., 2016). However, there are known limitations to using natural polymers as the foundation for delivery systems. For example, controlling the release of molecules from these polymers is challenging. Collagen is known to degrade rapidly *in vivo* through protease action (Friess, 1998); however, various chemical modifications—including cross-linking (Aframian et al., 2002; He et al., 2011; Oliveira et al., 2019) or combination with other compounds (e.g., composite materials) (Niu et al., 2009; Ding et al., 2016)—have enabled researchers to significantly prolong the degradation rates of these natural polymers. Other limitations of natural polymers include fabrication costs, batch variability, and harvesting (Vo et al., 2012).

#### Composite Nanostructured Delivery Systems

Composite materials are often developed to overcome specific limitations of a given material, such as those described above. The optimal properties of each individual material can be leveraged when combining multiple components into one delivery system. Contributing to the sustained release of osteogenic factors, which is critical for *in vivo* outcomes, composite materials provide additional functionality that can be used to fine-tune the temporal release profile of a given compound. Some common examples include ceramic/polymer composites, polymer blends, and silica/polymer composites. Several different polymer and HA composite materials for controlled delivery of BMP-2 have been described, including silk fibroin/poly(ethylene oxide)/nano-HA (Li et al., 2006), gelatin/fibrin/nano-HA (Liu et al., 2009), collagen/poly(L-lactic acid)/nano-HA (Niu et al., 2009), chitosan/nano-HA (Reves et al., 2011), and ε-polycaprolactone/HA (Kim B.-S. et al., 2018). The polymer components can be cross-linked and functionalized, whereas the ceramic components provide osteoconductive properties. Multiple polymers have been combined to create polymer blends, which provide further control of degradation rates (Wang et al., 2018). Metal/metalloid and polymer composites for drug delivery are a popular and expanding area of research in bone tissue engineering. Although metallic oxides like silica can be engineered to provide burst release of biomolecules, further functionalization with polymers can provide sustained release over time. Poly(lactic-co-glycolic acid)-mesoporous silicon composites have also gained significant traction as a delivery system. For example, PLGA-mesoporous silicon microspheres have been engineered to deliver therapeutics, including BMP-2 (Minardi et al., 2020) and other bioactive molecules (Minardi et al., 2015b); these have demonstrated excellent release profiles, biocompatibility, and osteogenic profiles both *in vitro* and *in vivo*. Other groups have utilized similar composite systems for bone tissue engineering applications (Fan et al., 2012; Zhang Q. et al., 2018). In another study, Singh et al. (2015) created a composite of PCL nanofibers coated with a mesoporous silica shell that was capable of binding to and providing sustained release of several bioactive molecules, with a subsequent upregulation of osteogenic differentiation *in vitro*.

### Biomaterials Functionalized With Nanostructured Delivery Systems

Scaffolds can be engineered to provide both spatially- and temporally controlled release of important biomolecules that facilitate bone regeneration and healing (Minardi et al., 2014, 2016b). While temporally controlled release is important to orchestrate the cascade of molecular and cellular events necessary to bone healing, the spatial release of biomolecules *in vivo* ensures that it occurs at the defect or desired area of interest for clinical applications (Minardi et al., 2016b). The most popular types of scaffolds (hydrogels (Nguyen and West, 2002), ceramics (Habraken et al., 2007), 3D-printed materials (Yi et al., 2016), and various composite materials (Yi et al., 2016) have all been proposed in combination with nanostructured delivery systems. These scaffolds can be functionalized with various bioactive components and molecules (Minardi et al., 2016b), including growth factors, peptides and mimicker molecules, immunomodulatory molecules, antibiotics, and even entire cells. Well-established as a potent stimulator of osteogenesis, incorporation of BMP-2 or BMP-2 mimetics into scaffolds is understandably an active area of research. Angiogenic factors such as VEGF, platelet derived growth factor (PDGF), and fibroblast growth factor (FGF) have also been shown to play an important role in bone regeneration and to support the maturation of the newly formed bone (David Roodman, 2003; Kaigler et al., 2006; De la Riva et al., 2010).

In addition to growth factors, systems can be functionalized to deliver other important bioactive molecules for bone healing, including immunomodulatory therapeutics and antibiotics to optimize bone healing. The host inflammatory response plays a critical role in osteogenesis and bone healing (Walsh et al., 2006; Guihard et al., 2012, 2015; Corradetti et al., 2015), and incorporation of immunomodulatory molecules within scaffolds provides another means to optimize bone healing and scaffold integration. Given that the risk of infection, including osteomyelitis, is significant after placement of implants (Lucke et al., 2003), the controlled release of antibiotics has the potential to provide a huge advantage to implanted devices and subsequent bone healing (Adams et al., 2009). Functionalization with these various molecules can occur via several different mechanisms, including incorporation of nanostructured systems within the scaffold, cross-linking and surface modifications, adsorption, direct loading of cells, among others. For example, various types of scaffolds—fibrous gelatin, poly(L-lactide), and HA particle composite (Amjadian et al., 2016) and electrospun nanofiber disks (Li et al., 2015)—have been functionalized to deliver local dexamethasone to improve osteogenesis. Herein, we list and review the most common and successful strategies for incorporation of delivery systems into 3-dimensional implants for bone regeneration.

#### Direct Incorporation of Nano-Delivery Systems in 3D Constructs

Direct incorporation of bioactive molecules and nanostructured delivery systems has been accomplished using a number of different techniques. One common modality is the hydrogel. Numerous polymeric hydrogels have been developed (Hoare and Kohane, 2008), as they can be engineered to both control the release of biomolecules (Gibbs et al., 2016) and enhance cellular adhesion and differentiation (Tibbitt and Anseth, 2009). These hydrogels are typically biocompatible and can be easily functionalized with cell adhesion ligands by modification of their surface (Hoffman, 2012). Some challenges persist, including their fabrication and clinical deployment (Hoare and Kohane, 2008). Hydrogels also inherently lack a solid framework, can be difficult to handle, and they may be difficult to sterilize, which can limit the clinical utility of these materials (Hoffman, 2012). Madl et al. (2014) engineered alginate hydrogels functionalized with a peptide mimetic of BMP-2, which were shown to upregulate markers of osteogenic differentiation and increase mineralization *in vitro*. Delivery systems of angiogenic factors such as VEGF have also been incorporated into polymeric hydrogel scaffolds for delivery *in vivo* (Kempen et al., 2009).

#### Surface Modification and Cross-Linking of Nano-Delivery Systems to 3D Constructs

Surface modification and cross-linking are other modes of biomaterials functionalization. Surface chemistries can facilitate stable, covalent binding of molecules, with the potential to provide tightly controlled release (Nie et al., 2007). Heparin-based linkers, for example, are commonly used to link growth factors to surfaces (Liang and Kiick, 2014). Various examples of these linkers have been described for local delivery of BMP-2 (Kim S.E. et al., 2011; Yun et al., 2013) and angiogenic factors like VEGF (Lode et al., 2008; Singh et al., 2011) and PDGF (Lee et al., 2012). Delivery systems of BMP-2 have also been incorporated directly onto the surface of 3D-printed ceramic scaffolds using polymer emulsion (Kim B.-S. et al., 2018).

Surface modification with immunomodulatory molecules has also been described. For example, Spiller et al. (2015) functionalized a decellularized scaffold with IL-4 via biotin-streptavidin binding and/or IFN-γ via adsorption, to facilitate rapid release of IFN-γ to promote pro-inflammatory M1 macrophages and sustained release of IL-4 to promote pro-healing M2 macrophages. Other groups have functionalized scaffolds with immunomodulatory molecules, including IL-4 (Minardi et al., 2016a), IL-10 (Rodell et al., 2015), and IL-33 (Liu et al., 2018) for various applications. The delivery of immunomodulatory molecules is relatively novel, particularly for bone tissue engineering, and this area of research is at a pivotal stage. Notably, scaffolds functionalized with IFN-γ have demonstrated increased vascularization relative to controls (Spiller et al., 2015).

Many other scaffolds have been engineered to provide a more sustained release of encapsulated BMP-2 (Yang et al., 2013; Rahman et al., 2014; Gan et al., 2015; Kim B.-S. et al., 2018; Mohammadi et al., 2018). Sun et al. (2018) fabricated a porous scaffold comprised of sintered HA nanoparticles, functionalized with either BMP-2 or BMP-2-related peptide, and both options provided significant osteogenic potential when assessed in a critical-size cranial defect model in rats. Hu et al. (2012) anodized a titanium substrate to form TiO_2_ nanotubes that were loaded with BMP-2 for sustained release, which showed promising upregulation of osteogenic differentiation *in vitro*.

#### Multifunctional Nanofiber Scaffolds as Drug Delivery Systems

Some nanostructured materials can simultaneously be used for the loading and release of bioactive molecules or to bind endogenous growth factors as well as to fabricate 3D scaffolds. A prime example is the use of peptide amphiphiles. Stupp and coworkers engineered a heparin-binding peptide amphiphile (HBPA) nanogel capable of binding and mimicking physiologic BMP-2 signaling (Lee et al., 2013). This BMP-2-binding PA promoted bone regeneration in a rat critical size femoral defect model with 10-fold lower doses than typically required (Lee et al., 2013). Additional approaches by this group have shown that BMP-2-binding PA nanogels provide significant bone regenerative capacity in an established pre-clinical posterolateral lumbar fusion (PLF) model with 10-fold lower doses of BMP-2 than typically required (Lee et al., 2015). In another study, hydrogels were designed with BMP-2 mimicking peptides that were capable of inducing osteogenic differentiation of rat MSCs *in vitro*. This osteogenic capacity was confirmed *in vivo* using a rat cranial defect model (Liang et al., 2019). In a recent study, peptide amphiphiles were functionalized with supramolecular glycopeptide nanostructures containing sulfated monosaccharides (Lee S. S. et al., 2017), given that heparan sulfate chains are a critical motif for the binding of many osteogenic growth factors under physiologic conditions (Xu and Esko, 2014). When assessed *in vivo* using an established rat PLF model, the PA nanostructures combined with a 100-fold lower dose of BMP-2 than typically required (100 ng of BMP-2 per animal) yielded an impressive 100% fusion rate (Lee S. S. et al., 2017). PA nanostructures combined with an even lower dose of BMP-2 (10 ng/animal, or 5 ng/scaffold) or without BMP-2 did not yield fusion, although PA nanofibers alone were minimally bioactive when assessed *in vitro* in C2C12 cells (Lee S. S. et al., 2017). The pre-clinical data regarding the use of PA-based materials for bone regeneration are promising. Although it is possible to control the nano-scale properties of the PAs, functionalize with different binding motifs, and gel the material into a macrostructure for *in vivo* applications, some future challenges include optimizing these materials for different clinical applications, ensuring minimal batch-to-batch variability, and large-scale fabrication of these materials.

These studies are but a few examples of the surface chemistries that can be employed to functionalize scaffolds with bioactive molecules. Given the multitude of variables that can be manipulated, the potential for drug delivery and bone regeneration applications are ever-expanding.

## Discussion and Conclusion

While each of the previously discussed approaches to bone regeneration show promise, bone defects are not all alike. For instance, the repair of large bone defects resulting from trauma requires a mechanically competent scaffold (Masquelet et al., 2000). Arthroplasty, on the other hand, calls for strategies that improve implant lifespan, as the longevity of conventional materials remains a major limitation in this setting (Smith et al., 2018). For this application, the key to success lies in improving the osseointegration of existing implants through surface modifications (Serra et al., 2013; Puertolas and Kurtz, 2014). Orthopedic infection is another major challenge to implant-based bone healing, and additional material characteristics, such as antimicrobial properties, should be considered.

Even so, the biological environment for bone healing may also be compromised as a result of numerous patient-related factors, local and/or systemic, including advanced age, gender, tobacco, and/or alcohol use, pre-existing chronic illness, and the use of certain medications (Andrzejowski and Giannoudis, 2019). For example, the bone healing deficiencies observed in older patients or smokers may require cell-based approaches or growth factors such as rhBMP-2, which may reliably stimulate healing but can be associated with significant adverse effects. Another example is the use of biologics, which is a strategy of choice in several orthopedic procedures, but that remains inappropriate in oncologic patients, where this may potentially exert local or even systemic tumor-promoting effects (Serakinci et al., 2014; Holzapfel et al., 2016). Together, these influences act through a variety of mechanisms to predispose some patients to impaired bone regeneration, which can only be overcome by personalized regenerative strategies. By providing more precise and individualized treatment modalities, nanotechnological approaches to bone regeneration may provide more effective and longer-lasting implants, decreased infection rates, and improved bony healing, which could ultimately translate to improved patient outcomes. In particular, nanotechnology has allowed for the design of materials that can approach the challenge of augmenting bone regeneration from different angles, such as simultaneously mimicking the bone nano-composition and structure, while serving as a delivery vehicle for bioactive molecules and/or cells (Zhao et al., 2011). Nano-biomaterials that are able to recapitulate more than one of the aspects of bone as reviewed herein may certainly offer superior performances in challenging clinical settings. For example, nanotechnology offers oncologic patients novel means of integrating drug delivery functions into osteoinductive biomaterials which in turn can be used for both the regeneration of bone defects as well as the targeted treatment of the cancer (Acharya and Sahoo, 2011; Gu et al., 2013; Rawat et al., 2015).

Given that each bone defect and combination of pre-existing conditions may call for different regenerative strategies or a combination of them, it is also crucial not to overlook the role of the host’s immune system in bone healing. In fact, the immune system not only protects the body from pathogens but also orchestrates the response to foreign materials, and monitors for possible alterations in tissue homeostasis through a mechanism known as *inflammation* (Taraballi et al., 2018). While many researchers have investigated methods to minimize the immune response to materials to preserve their regenerative potential, in recent years there has been a shift toward the development of technologies able to preferentially engage the host’s immune cells. In fact, inflammation and the subsequent recruitment of immune cells to the diseases site are paramount to initiate healing (Taraballi et al., 2018). Nonetheless, while inflammation is desirable due to its key role in initiating tissue repair, it does need to be controlled in order to avoid the initiation of a foreign body response against bone regenerative materials (Franz et al., 2011).

**FIGURE 4:**
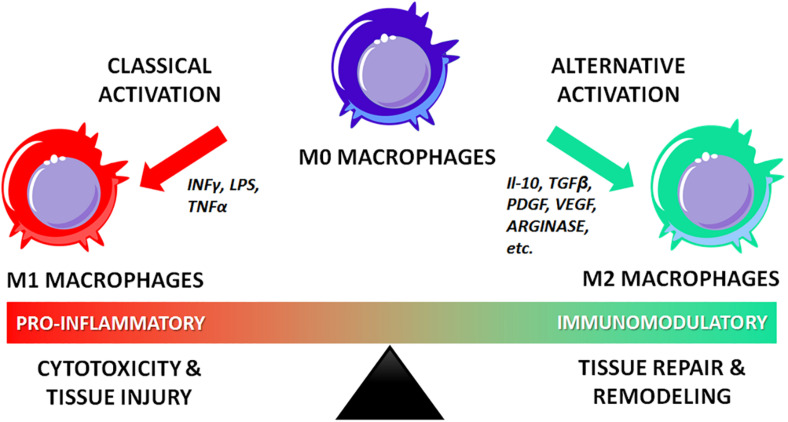
Role of classically and alternatively activated macrophages in inflammation, tissue injury and regeneration.

Inflammation consists of the infiltration, proliferation, and polarization of hematopoietic and non-hematopoietic cells, that are recruited and activated by specific bioactive factors produced within the lesion (Taraballi et al., 2018). Among the cells involved in this highly orchestrated process, macrophages have been found to be the primary players (Wynn and Vannella, 2016; Michalski and McCauley, 2017). Classically activated macrophages (M1) are the first to be recruited to the site of injury and are gradually replaced by the alternatively activated macrophages (M2) if a regenerative response is initiated (Minardi et al., 2016a). M2 macrophages are immunomodulatory and coordinate tissue repair-producing anti-inflammatory molecules such as IL-10 and TGF-β; this triggers angiogenesis and matrix remodeling, while suppressing the M1-mediated inflammation (Figure 4; Minardi et al., 2016a).

Currently, the two main strategies to modulate the macrophage-driven inflammatory response are through (i) materials functionalized with nanodelivery systems for the release of immunomodulatory mediators (Spiller et al., 2015); (ii) materials engineered at the nanoscale so that their composition and structure itself may induce macrophage polarization toward the M2 lineage (Vasconcelos et al., 2016; Zhang R. et al., 2018; Lee et al., 2019). Compared to classical cell-based regenerative strategies, immunomodulatory strategies leverage on the self-healing capabilities of the body, thus resulting less technically challenging, since they do not require the direct encapsulation or delivery of live cells.

Although the field of immunomodulatory materials is still in its relative infancy, nanostructured materials have proven to have the necessary level of sophistication to address the challenges of this new arena. Nanostructured immunomodulatory materials will be amongst the most disruptive bone regenerative technologies, as the future of bone regeneration is clearly headed toward increasingly personalized approaches.

## Author Contributions

SM, EH, and WH conceived and outlined the review. All authors wrote and edited the manuscript and designed the schematics.

## Conflict of Interest

The authors declare that the research was conducted in the absence of any commercial or financial relationships that could be construed as a potential conflict of interest.
